# The March to the Grave

**Published:** 1854-07

**Authors:** 


THE MARCH TO THE GRAVE.—What a mighty procession has been marching towards the grave during the past year! At the usual estimate, since the 1st of January, 1853, more than 31,500,000 of the world's population have gone down to the earth again. Place them in a long array, and they will give a moving column of more than thirteen hundred to every mile of the globe's circumference! Only think of it; ponder and look upon these astounding computations! What a spectacle, as they "move on," tramp, tramp, tramp—forward upon this stupendous dead march!

> Life is short and time is fleeting,
> And our hearts, though strong and brave,
> Still, like muffled drums, are beating
> Funeral marches to the grave.

